# No preference for direct versus averted gaze in autistic adults: a reinforced preferential looking paradigm

**DOI:** 10.1186/s13229-020-00398-3

**Published:** 2020-11-18

**Authors:** Elise Clin, Pauline Maes, Fanny Stercq, Mikhail Kissine

**Affiliations:** ACTE at LaDisco and ULB Neuroscience Institute, Université libre de Bruxelles, Avenue F. D. Roosevelt, 50/175, 1050 Brussels, Belgium

**Keywords:** Autism, Eye-tracking, Eye gaze direction, Social attention, Alexithymia, Social anxiety, Gender, Adults, Reinforced preferential looking paradigm

## Abstract

**Background:**

With the overarching objective to gain better insights into social attention in autistic adults, the present study addresses three outstanding issues about face processing in autism. First, do autistic adults display a preference for mouths over eyes; second, do they avoid direct gaze; third, is atypical visual exploration of faces in autism mediated by gender, social anxiety or alexithymia?

**Methods:**

We used a novel reinforced preferential looking paradigm with a group of autistic adults (*n* = 43, 23 women) pairwise matched on age with neurotypical participants (*n* = 43, 21 women). Participants watched 28 different pairs of 5 s video recordings of a speaking person: the two videos, simultaneously displayed on the screen, were identical except that gaze was directed at the camera in one video and averted in the other. After a 680 ms transition phase, a short reinforcement animation appeared on the side that had displayed the direct gaze.

**Results:**

None of the groups showed a preference for mouths over eyes. However, neurotypical participants fixated significantly more the stimuli with direct gaze, while no such preference emerged in autistic participants. As the experiment progressed, neurotypical participants also increasingly anticipated the appearance of the reinforcement, based on the location of the stimulus with the direct gaze, while no such anticipation emerged in autistic participants.

**Limitations:**

Our autistic participants scored higher on the social anxiety and alexithymia questionnaires than neurotypicals. Future studies should match neurotypical and autistic participants on social anxiety and alexithymia and complement questionnaires with physiological measures of anxiety.

**Conclusions:**

The absence of preference for direct versus averted gaze in the autistic group is probably due to difficulties in distinguishing eye gaze direction, potentially linked to a reduced spontaneous exploration or avoidance of the eye region. Social attention and preference for direct versus averted gaze correlated with alexithymia and social anxiety scores, but not gender.

## Background

Atypically low attention to other people’s faces—and, more particularly, to the eye region—is one of the most documented diagnostic criteria for autism spectrum disorder [1: 50], which is used in screening tools [2] and diagnostic assessments [3]. Facial expression and gaze play a crucial role in several areas that are known to be affected in autism: language development [4], joint attention [5], face and emotion recognition [6], mentalizing [7] and conversation management [8]. Yet, despite substantial research on social processing in autism, three crucial issues about the way autistic adults^1^ explore faces remain outstanding: (a) Do autistic adults prefer looking at mouths over eyes?; (b) do autistic adults avoid direct eye gaze?; and (c) do individual characteristics, such as gender, social anxiety or alexithymia, mediate atypical visual exploration of faces in autistic adults?

### Do autistic adults prefer mouths over eyes?

In a seminal eye-tracking study, Klin et al. [10] found that, in watching excerpts from a movie, neurotypical adults preferentially looked at the protagonists’ eyes, whereas autistic participants looked less at faces, and, when they did, mostly paid attention to the mouth region. Klin et al. [10] hypothesize that autistic adults preferentially gaze at mouths of speaking people, because this is the facial region that provides them with essential interactional information.

However, it is unclear whether autistic adults genuinely prefer the mouth region or whether they simply avoid the eye region [11, 12]. While some studies reported a preference for mouths over eyes in autistic adults [11–17], others did not replicate this preference [18–24] or even found no group differences in fixations on the eyes [25, 26] or on the mouth [16, 27–30]. Such inconsistent results may be partly due to methodological variation between studies: some paradigms render the mouth region salient [e.g. using complex dialogues, as in 10], while others attract participants’ attention to the eyes [e.g. with explicit instructions, as in 26]. A promising way to determine the extent to which autistic adults are genuinely attracted to mouths would be to render the eye region particularly relevant, while keeping the mouth region salient. If the preference for the mouth is robust, one should expect it to obfuscate the relevance of the eyes to autistic participants.

### Do autistic adults avoid direct eye gaze?

An important feature of the studies that found a higher amount of fixation on the mouth region in autistic adults is that they all used stimuli with direct eye gaze [11–17]. Even though one can assume that diverse mechanisms operate across the whole autism spectrum, it does make sense to hypothesize that a significant number of autistic people avoid eye contact—rather than the eye region per se—because eye contact may provoke an excessive emotional arousal [as suggested by the hyperarousal model, in opposition to hypoarousal; see 31]. Several studies found that autistic adults are able to detect [32, 33] and follow [34, 35] eye gaze direction. However, difficulties in detecting gaze direction have also been documented [36], especially with subtly averted gazes [37, 38]. Neurotypical adults are known to display a preference for direct versus averted gaze [39–41]. Two eye-tracking studies, using an interocular suppression paradigm [39], directly tested unconscious preference for direct versus averted gaze in autistic adults [40, 42], and none found a preference for direct gaze. However, these studies relied on repeated and silent presentation of still, greyscaled pictures of identical female faces. Such stimuli are quite removed from the actual experience of attending to faces, contrary to the growing trend in the literature on social attention to strive for ecological validity in stimulus design [see 43]. In this study, we sought to determine whether autistic adults avoid the eye region specifically when gaze is fixated on them, or if they do not attend to it in any case. In each trial, we simultaneously presented autistic people with two identical colour videos of different speaking faces, one with eyes directed at the camera and one with averted eyes.

### Do social gender, social anxiety or alexithymia contribute to atypical processing of eyes in autism?

While there is a growing interest in gender differences in autism, only a few eye-tracking studies controlled for the effects of gender on social processing in autism [38, 44–49]. Most of these studies (mainly based on child samples) suggest a potentially higher social attention in autistic females compared to autistic males. It is therefore important to include gender-balanced samples when studying visual exploration of faces in autistic adults.

Social anxiety, defined as a fear of negative evaluation that leads to an excessive concern about social situations [1: 202–203], is a comorbidity often attested in autistic adults [50–52]. Socially anxious individuals may look less at faces and avoid direct gaze [53–59, but see 60]. For this reason, it is important to control for social anxiety in investigating social cue processing in autism.

Finally, alexithymia is a personality trait that could also impact the processing of social cues [61]. Alexithymia is defined as a difficulty to identify and label emotions and is often attested in autistic individuals [62]. For example, in Bird et al. [63], alexithymia scores, but not autism severity, significantly predicted eye-to-mouth fixation ratios. Unfortunately, alexithymia is rarely measured in studies assessing social attention (in both neurotypical and autistic populations).

### Current study

We designed a reinforced preferential looking paradigm, in which participants are simultaneously presented with two almost identical videos of speaking faces. These videos differ in only one respect: one displays a direct gaze, whereas the other displays an averted gaze. The video with direct eye gaze is always reinforced with a short amusing animation. Implicitly learning, over the course of the trials, the association between the video with the direct gaze and the reinforcement animation thus requires distinguishing between direct and averted gaze. This reinforcement makes the eyes the most relevant region of our stimuli. Accordingly, unless they are intrinsically attracted to the mouth region, autistic participants should not look more at the mouth than at the eyes. While we expect neurotypical adults to display a preference for direct gaze, two concurrent predictions may be made about the impact of the reinforcement of the direct gaze on autistic participants. On the one hand, if autistic participants do discriminate between direct and averted gaze, they should fixate more direct gaze stimuli over the course of the trials; on the other hand, if autistic participants do not distinguish direct from averted gaze—because of difficulties in discriminating eye gaze direction or of an overall lower exploration of the eye region (due to a lack of interest, avoidance or both)—they should not display any preference for direct gaze videos. Finally, we composed gender-balanced samples and assessed social anxiety and alexithymia in our participants. We expect more social attention in autistic females compared to autistic males. We also expect high social anxiety or alexithymia scores to correlate with reduced attention to the eye region.

## Methods

### Eye-tracking task

**Fig. 1:**
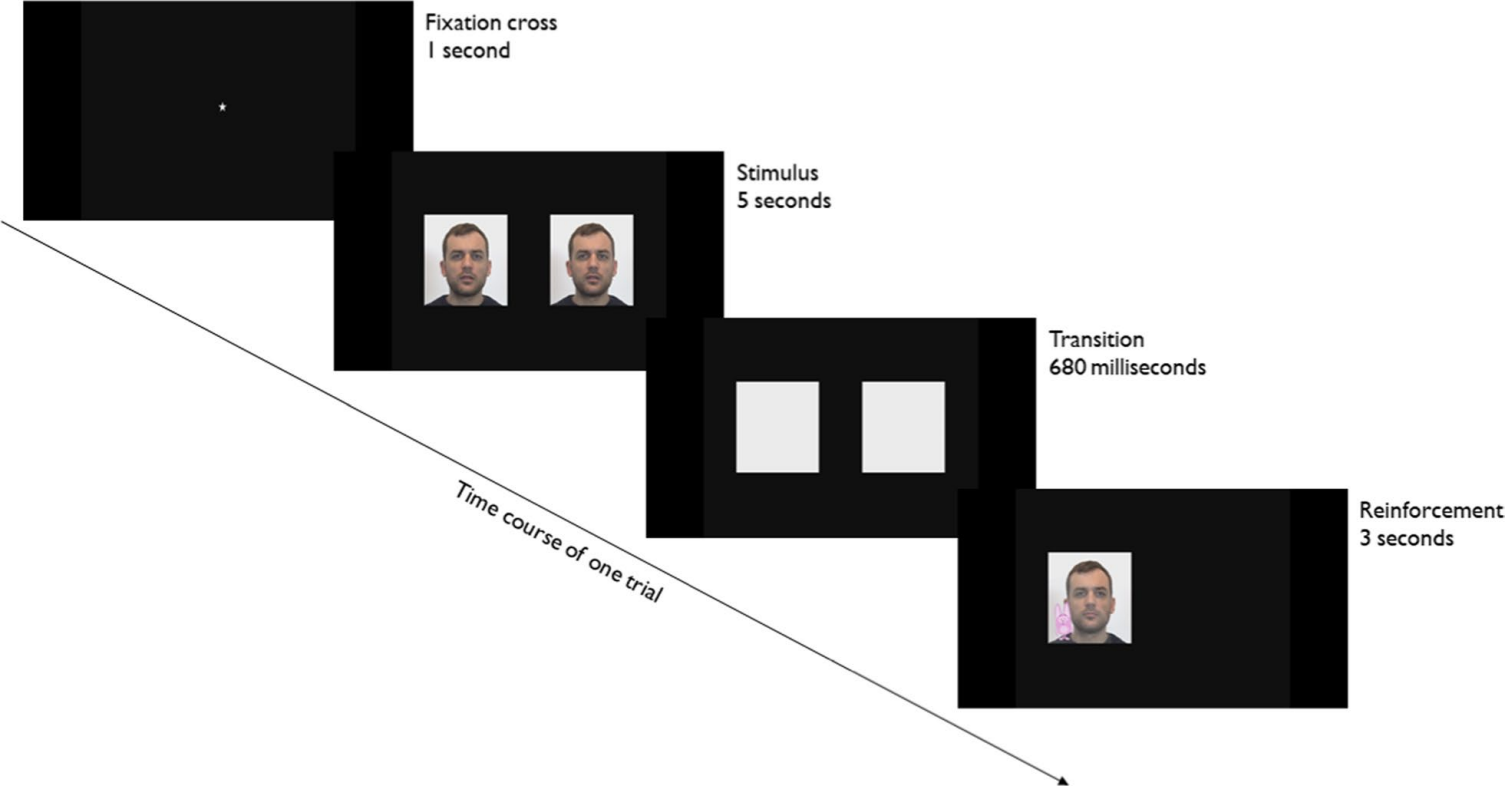
Eye-tracking task. Time course of a trial

As depicted in Fig. 1, each trial consisted of a 1-s fixation cross, a 5-s stimulus, a 680-ms transition window and, finally, a 3-s reinforcement phase.

**Fig. 2:**
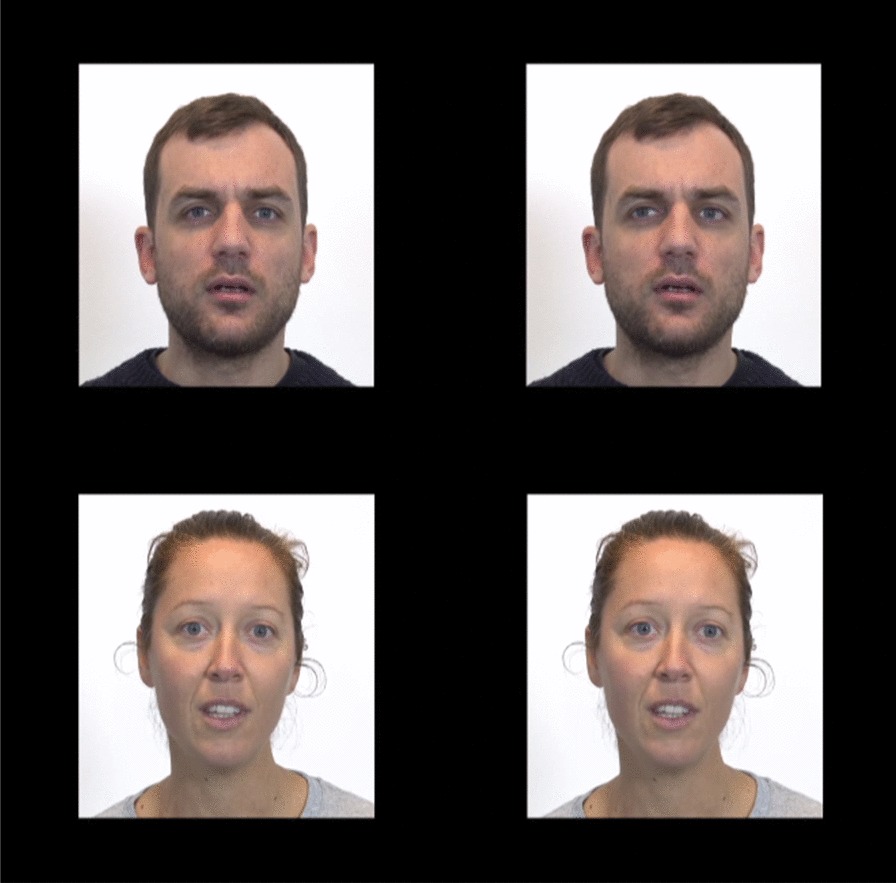
Eye-tracking task. Top pane: on the left, the original video, with direct gaze, and, on the right, the modified version of this video, with an obvious averted gaze directed to the left. Bottom pane: on the left, the original video, with direct gaze, and, on the right, the modified version of this video, with a subtle averted gaze directed to the right

Each trial began with a fixation cross, displayed in the centre of the screen along with a short jingle to maintain the participant’s attention. Each stimulus was 5-s long and consisted of two colour videos. As shown in Fig. 2, these two videos were simultaneously displayed side by side and were identical except for the direction of the actor’s gaze. In one video (the original one), the actor’s eyes were directed right at the camera; in the other video, gaze direction was artificially modified, so that the eyes were averted either to the right or to the left. The stimulus presentation phase was followed by a 680-ms transition phase, during which the two videos were replaced, in exactly the same positions, by two grey squares. In each trial, the stimulus with direct gaze was next reinforced by a funny animation. These reinforcement videos consisted of a snapshot of the face of the actor from the direct gaze stimulus video with a drawing of an imaginary animal gradually appearing either on the right or left shoulder of the person. The main purpose of the transition phase thus was to allow the participant to anticipate the appearance of the reinforcement.

Our stimuli differ in several respects from those used in previous studies that simultaneously presented direct versus averted gaze in the autistic population [36, 40, 64]. First, to increase ecological validity, we used colour videos with soundtracks and gender-counterbalanced actors instead of still photographs. At the same time, to avoid information overload in autistic participants, we minimized stimulus complexity, having one actor at a time and suppressing any background information [see 65]. Second, to focus on the processing of gaze direction, we used two perfectly identical stimuli which differed only in eye gaze direction. Third, we used speaking stimuli which likely prompt an increased visual exploration of faces [66, 67, but see 68].

Gaze direction of the original videos was modified by a special effects expert using Adobe After Effect, based on two factors: intensity of the deviation angle and direction (right or left). Intensity of gaze deviation was either subtle, resulting in a slightly averted gaze (*M*: 9.36°; SD: 1.86; range 5–12), or obvious, resulting in a more strongly averted gaze (*M*: 18.21°; SD: 2.39; range 13–22). Three independent non-autistic judges (one woman) were asked to watch the videos and signal any unnatural item. Based on their comments, the most convincing items were selected (one averted gaze per actor, either subtle or obvious). None of the judges guessed that the gazes had been artificially modified. Four different types of averted stimuli were eventually counterbalanced across trials: (1) subtle gaze diversion to the right; (2) subtle gaze diversion to the left; (3) obvious gaze diversion to the right; and (4) obvious gaze diversion to the left. The original video was presented on the right side of the screen and the modified video on the left side of the screen in one half of trials, and vice versa in the other half.

The French sentences uttered by the actors in the video stimuli, one per stimulus, were created using the database Lexique3 [69]. They all followed the same grammatical structure (see Additional file 1). The actors were instructed to display the most neutral facial expression possible, as smiling faces may increase the impression of direct gaze, even though the eyes are in fact averted [37].

A total of 28 different trials were presented to each participant (for a total duration of 5 min). Trials were pseudo-randomized in such a way that direct gaze stimulus would not appear on the same side of the screen more than three times in a row, that the averted eyes were not looking in the same direction more than three times in a row, and that the averted gaze was not of the same intensity more than three times in row.

Eye movements were recorded at 60 Hz using a Tobii Pro × 2–60 remote eye-tracker. Stimuli were displayed on a 1920 × 1080 computer screen. The eye-tracker was located just underneath the screen. Participants were seated approximately 60 cm away from the screen to ensure optimal measures. To avoid any instruction bias [see 70], we simply told our participants to carefully look at the screen without moving or speaking. Prior to the task, participants underwent a standard eye-tracking nine-point calibration procedure.

### Participants

The autistic group was composed of 43 adults (23 women), aged 19–55 years (*M* = 35.79; SD = 9.98). Autistic participants were matched pairwise by age (age difference in months: *M* = 23; SD = 13.63), and groupwise by full-scale (FIQ) and verbal intelligence quotients (VIQ) with a neurotypical group consisting of 43 adults (21 women), aged 21–58 years (*M* = 35.56; SD = 11.4). Participants were asked about their affiliated gender and one autistic participant reported to be non-binary: she is anatomically a man but socially identifies herself as a woman (those are her own terms). We decided to respect this identification and included her in the women group [see 71 for a discussion on autism and the non-binary population]. Participants were recruited through our laboratory database, flyers (published on social media or pinned in public places) and personal networks. Inclusion criteria for autistic participants were being a native French speaker, being verbally fluent, having normal or corrected-to-normal vision and audition, and no intellectual delay. All autistic participants received a clinical diagnosis of autism or Asperger syndrome from officially habilitated multidisciplinary teams, based on the Autism Diagnostic Observation Schedule (ADOS) [3] and the Autism Diagnostic Interview-Revised (ADI-R) [72] criteria. Four additional autistic participants were not included in the final data set because their diagnosis has not been confirmed by a multidisciplinary team. For neurotypical participants, inclusion criteria were being a native French speaker, having normal or corrected-to-normal vision and audition, and no history of developmental delays, psychiatric diagnoses or neurocognitive impairments. We did not attempt to match our groups on socio-economic variables: autistic people, even if they are intellectually able, often encounter difficulties in their academic and working lives because of their autism, which can negatively impact their socio-economic status [73, 74]. In both groups, there was a minority of childhood bilinguals (10 in the autistic group; 11 in the neurotypical group). Participant characteristics are summarized in Table 1.

**Table 1:** Descriptive statistics for groups

Measures	Autistic group (*n* = 43; *F* = 23)		Neurotypical group (*n* = 43; *F* = 21)		*t* test
	*M*	SD	*M*	SD	
Age (years)	35.79	9.98	35.56	11.4	− 0.101
Full-scale IQ	119	15.27	118.53	9.47	− 0.170
Verbal IQ	125.07	15.41	126.86	12.74	0.581
Economic status	6.05	2.08	7.40	1.90	3.115**
Level of education	3.26	1.38	3.70	1.06	1.636
Autism quotient	38.81	5.34	16.84	5.96	− 18.006***
Empathy quotient	20.53	8.42	43.93	10.37	11.484***
Social anxiety	76	31.14	30.77	25.13	− 8.584***
Alexithymia	61.77	10.85	45.81	10.15	− 6.735***

Missing data: 2 verbal intelligence quotients (autistic group); 1 economic status and level of education (autistic group); 8 social anxiety and alexithymia questionnaires (autistic group: 2; neurotypical group: 6)

*p < 0.05; **p < 0.01; ***p < 0.001

### Intelligence quotient

Participants’ intelligence quotients were assessed using the Wechsler Adult Intelligence Scale IV [75]. The WAIS-IV is composed of 10 core subtests which yields 4 indexes: verbal comprehension, perceptual reasoning, working memory and processing speed. The full-scale IQ combines those indexes.

### Questionnaires

Participants were asked to complete five predesigned self-administered questionnaires. Our laboratory questionnaire, adapted from the revised Family Affluence Scale [76–79], provides a proxy for the participant’s socio-economic background: the education score is a 0-to-6-point scale (0 being no primary school achieved; 6 being the doctoral degree), and the economic status score is a 0-to-13-point scale (0 being very low; 13 being very high); it was also used to determine our participants’ bilingualism history, as well as their personal and family medical history. The Adult Autism Spectrum Quotient [80] and the Cambridge Behaviour Scale [81], assessing the empathy quotient, were also administered. A high number of autistic (AQ) traits (≥ 32), associated with low levels of self-reported empathy (EQ), are considered typical of autism [81]. Participants also filled in the Liebowitz Social Anxiety Scale [82], on which respondents are asked to rate one's fear and avoidance in front of different situations (e.g. answering a phone call in public). This scale’s outcomes are grouped in three different levels of social anxiety: mild (≤ 51), moderate (52–81) or severe (≥ 82). Finally, we measured alexithymia with the 20-item Toronto Alexithymia Scale—TAS-20 [83]. The TAS-20 is designed to measure the three components of alexithymia: difficulty identifying feelings in the self; difficulty describing feelings; and externally orientated thinking. This scale outcomes are grouped in three levels: not alexithymic (≤ 51), potentially alexithymic (52–60) and alexithymic (≥ 61). To date, the TAS-20 remains the most reliable scale to assess alexithymia, including in clinical samples [84], which correlates with other measures of alexithymia in autistic samples [e.g. 63].

### Experimental setting

Participants gave their written consent to be involved in this study after having been informed of their rights and all aspects of the sessions (number, length, content and collected data). All participants were individually evaluated by the first author or a trained master student in Neuropsychology. In order to maximize data quality, participants were encouraged to come to the laboratory, and most did (*n* = 59). However, some participants could not visit our laboratory, for personal or practical reasons (having no car, not being comfortable with public transportation, fearing a long trip, having time constraints or feeling overwhelmed in unknown places). In those cases, participants were tested at their home (*n* = 26) or office (*n* = 1), in a quiet and comfortable room, with a table (to put the computer or the WAIS-IV on) and two chairs. No session has been interrupted by an external event. The first session was composed of several tasks not reported here (word definition, executive functions and irony detection) and the eye-tracking task. For some participants, this session was split into two, because of scheduling conflicts or fatigue. When no valid IQ score was available (scores older than 1 year or IQ assessed by another scale than the WAIS-IV), the IQ test was administered during a second session.

### Data analysis

#### Eye-tracking data preparation

**Fig. 3:**
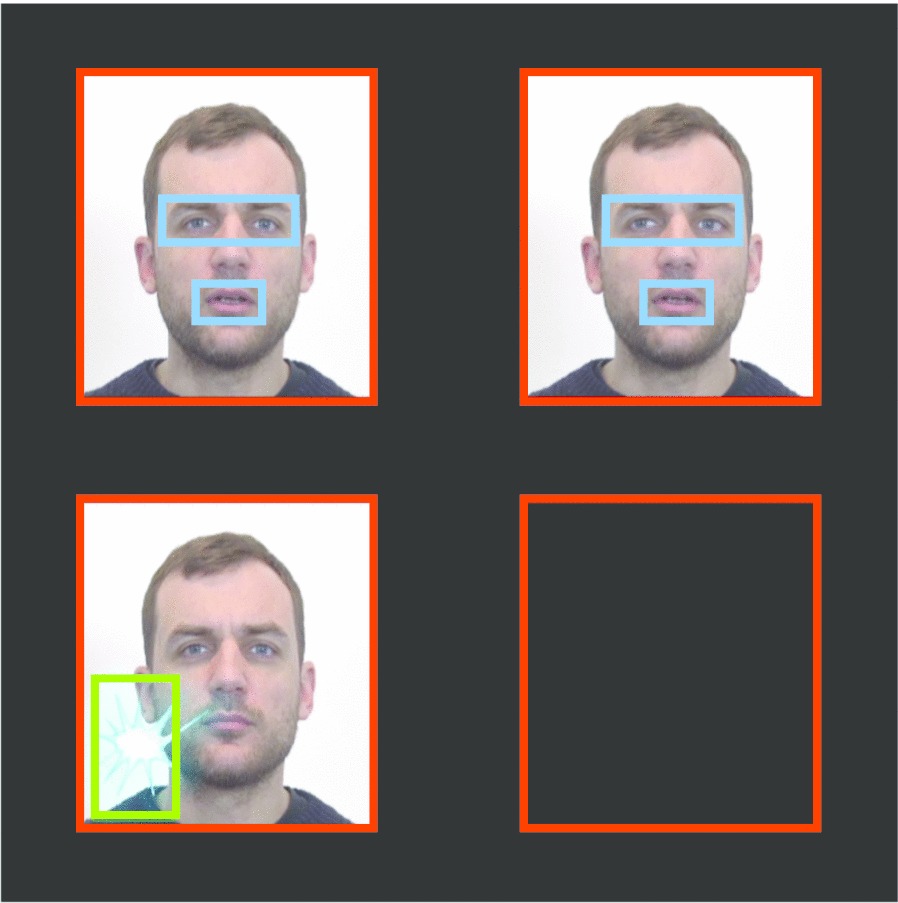
Areas of interest. Throughout the trial: left versus right squares. During the stimulus phase: eyes and mouths of the actor. During the reinforcement phase: reinforcement animation appearing on the direct eye gaze side

Several areas of interest (AOIs) were designed using the Areas of Interest tool of the Tobii Studio TM 3.4.5 Software (see Fig. 3). The first two AOIs were squares corresponding to the exact zones of the screen where the stimulus videos were displayed, one on the left and one on the right. These AOIs were identical for every trial. Additionally, for each trial, two specific AOIs were defined on the eye (including the eyebrows) and mouth regions: these AOIs were first drawn on the left video and then copied and pasted on the right video to ensure that the AOIs were identical for the two videos. Finally, for each trial an extra AOI was created for the exact zone where the animation appeared during the reinforcement phase. After correction for a potential calibration error (see Additional file 1), we used the Data Export tool of the Tobii Studio TM 3.4.5 Software to extract the eye-tracking data every 16 ms, resulting in a binomial variable which indicated for each AOI whether a fixation had been recorded or not. Finally, these values were aggregated over 500 ms intervals.

#### Statistical methods

All statistical analyses were implemented in R [85]. The following variables were used in the analysis: group (autistic versus neurotypical), stimulus type (direct versus averted gaze), subtlety (subtle versus obvious averted gaze), gender (male versus female), time (corresponding to 500 ms intervals), trial and, finally, social anxiety, alexithymia, full-scale intelligence quotient (IQ), autism quotient (AQ) and empathy quotient (EQ) scores. Aggregated fixations over 500 ms intervals were analysed with multilevel linear regressions using the lme4 package [86]. Post hoc comparisons of least-square means were carried out with the lsmeans package [87] with Tukey adjustment for multiple comparisons.

## Results

### Mouths versus eyes

**Fig. 4:**
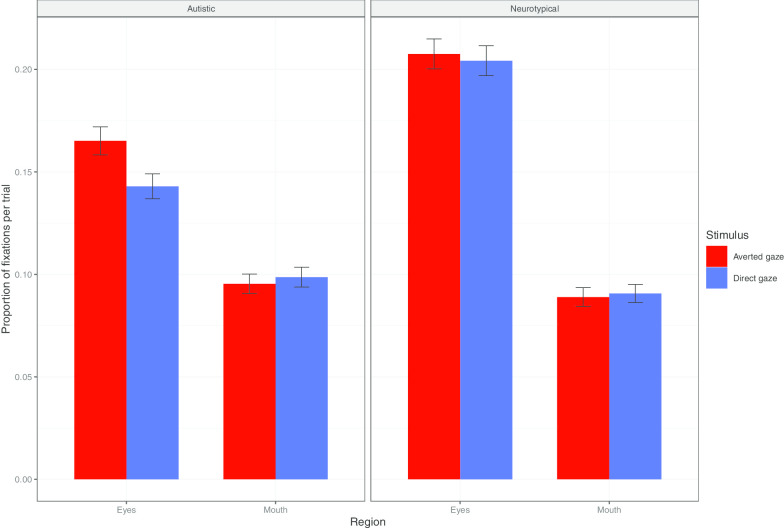
Stimulus presentation phase. Proportion of fixations on mouth versus eyes over the sum of fixations on each stimulus, per trial, by group. Vertical lines represent standard errors of means

To test the presence of a preference for the mouth region in our autistic participants, we computed, for each trial and each stimulus, the proportion of fixations on the eye and mouth regions over the total number of fixations (see Fig. 4). Stepwise comparisons of multilevel models, with by item and by participant random intercepts, indicated that the addition of Region significantly increased the model fit (*χ*^(1)^ = 514.86, *p* < 0.001), as also did the addition of the Region × Group interaction (*χ*^(2)^ = 63.78, *p* < 0.001). However, the Region × Stimulus Type and the Region × Stimulus Type × Group interactions did not prove significant (both p > 0.064). Post hoc comparisons indicated that, in both groups, the eye region attracted more fixations than the mouth region (neurotypical group: *β* = 0.12, SE = 0.5e^−2^, *p* < 0.001; autistic: *β* = 0.06, SE = 0.5e^−2^, *p* < 0.001). The eye region attracted more fixations in neurotypicals than in autistic participants (*β* = 0.55e^−1^, SE = 0.02, *p* = 0.026). By contrast, there was no group difference with respect to fixations on the mouth region (all *p* = 1). In sum, irrespective of the stimulus gaze direction, autistic participants fixated less the eye region, but did not display preferential attention to the mouth region.

### Direct versus averted gaze

Next, we investigated preferences for direct and averted gaze during the stimulus presentation phase. We ran stepwise comparisons of multilevel linear models, with by item and by participant random intercepts: the addition of Stimulus Type significantly improved the model fit (*χ*^(1)^ = 288.9, *p* < 0.001), as did the Stimulus Type × Group interaction (*χ*^(2)^ = 228.4, *p* < 0.001). By contrast, neither the Subtlety × Stimulus Type (*p* = 0.06) nor the Subtlety × Group × Stimulus Type interactions improved the model fit (*p* = 0.1). Post hoc pairwise comparisons indicated that neurotypical participants were more likely to look at the stimuli with direct gaze than at the stimuli with averted gaze (*β* = 0.14, SE = 0.6e^−2^; *p* < 0.001), while no such difference emerged in the autistic group (*p* = 0.36). Neurotypical participants were also more likely to fixate the stimuli with direct gaze than autistic participants (*β* = 0.1, SE = 0.23e^−1^; *p* < 0.001), but no group difference emerged relative to the amount of fixation on stimuli with averted gaze (*p* = 0.68).

**Fig. 5:**
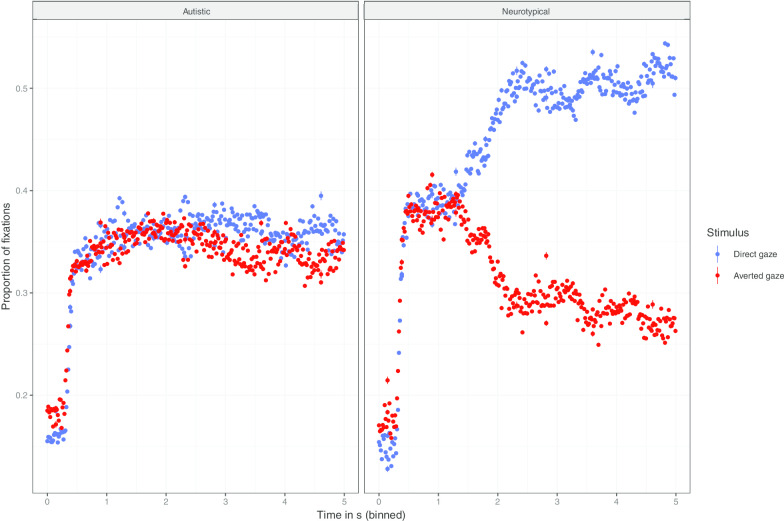
Stimulus presentation phase. Mean proportion of fixations during the stimulus presentation per time point, by group and stimulus type. Vertical lines represent standard errors of means

Figure 5 displays the curve of mean fixations, per group, during the stimulus presentation phase. These curves suggest that after an initial exploration of the two types of stimuli, neurotypical participants quickly focus on the face with direct gaze, contrary to autistic participants. In order to assess whether the looking patterns diverged between groups over the stimulus time course, we created a linear regression model predicting fixations on the stimulus with direct gaze, with group as a fixed factor and time by item and by participant random slopes. The addition of the time fixed factor significantly improved the model fit (*χ*^(1)^ = 27, *p* < 0.001), as did the Time × Group interaction (*χ*^(1)^ = 10.79, *p* = 0.001). Conforming to the visual impression in Fig. 5, the time slope for fixations on the stimuli with direct gaze was significantly higher in the neurotypical than in the autistic group (*β* = 0.03, SE = 0.01; *p* = 0.001).

**Fig. 6:**
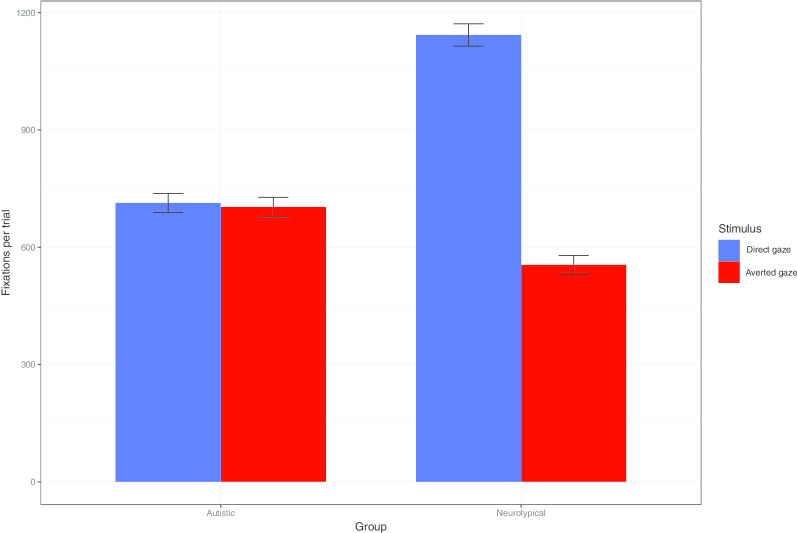
Transition phase. Average number of fixation points per trial, by stimulus type and group. Vertical lines represent standard errors of means

To further investigate this preference for direct gaze displayed by the neurotypical group, we analysed the average fixations per stimulus type and group during the transition phase (see Fig. 6). Aggregated fixations over 500 ms intervals were analysed using multilevel linear regressions, with by item and by participant random intercepts. Stepwise comparisons indicated that the addition of stimulus type significantly improved the model fit (*χ*^*(1)*^ = 493.74, *p* < 0*.*001), as did the Stimulus Type × Group interaction (*χ*^(2)^ = 484.13, *p* < 0*.*001). The Subtlety × Stimulus Type interaction did not improve the model fit (*p* = 0*.*52). Post hoc comparisons revealed that neurotypical participants preferentially gazed towards the area of the screen on which was displayed the stimuli with direct gaze (hereafter: direct gaze area), relative to the one on which was displayed the stimuli with averted gaze (hereafter: averted gaze area) (*β* = 0.24, SE = 0.7e^−2^; *p* < 0*.*001); by contrast, no such difference emerged in the autistic group (*p* = 0*.*92). Neurotypical participants also displayed more fixations on the direct gaze area than autistic participants (*β* = 0.17, SE = 0.22e^−1^; *p* < 0*.*001); on the contrary, autistic participants displayed more fixations on the averted gaze area than neurotypical participants (*β* = 0.68e^−1^, SE = 0.23e^−1^; *p* = 0*.*02).

Preferential fixations, during the transition phase, on the direct gaze area can be due to a spillover effect from the stimulus presentation phase or to the anticipation of the animated reinforcement, which always appeared on that side. We tested the presence of such an anticipation effect in two ways.

**Fig. 7:**
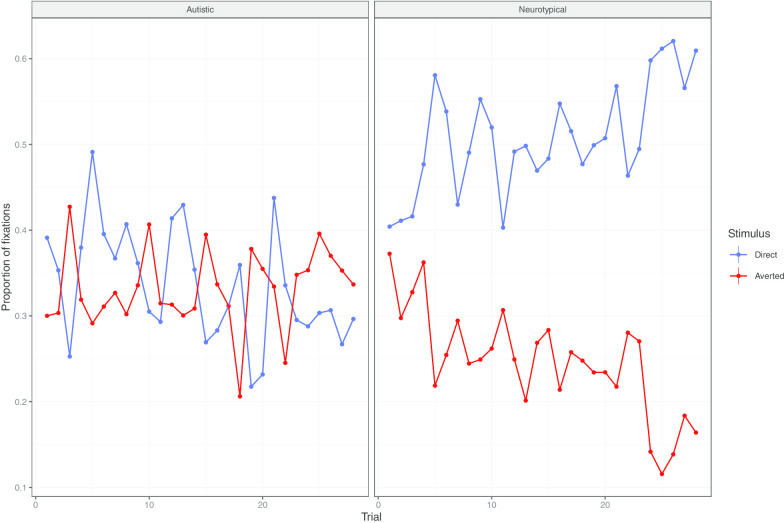
Transition phase. Mean fixation per trial, by group

First, an anticipation effect would depend on implicit learning of the reinforcement placement, which should emerge over the course of the trials. As can be seen in Fig. 7, in the neurotypical group, fixations on the direct gaze area increase over the course of the trials. The Stimulus Type × Group × Trial interaction significantly improved the fit of the model with Stimulus Type and Stimulus Type × Group fixed effects and with Trial by item random slopes and by participant random intercepts (*χ*^(2)^ = 134.86, *p* < 0.001; the model with trial by participant random slopes failed to converge). Post hoc analyses confirmed that, in the neurotypical group, the trial slope was positive for the fixations on the direct gaze area (*β* = 0.5e^−2^, SE = 0.1e^−2^), but negative for fixations on the averted gaze area (*β* = − 0.6e^−2^, SE = 0.1e^−2^), this difference being significant (*p* < 0.001). By contrast, in autistic participants, the trial slope was negative both for the fixations on the direct gaze area (*β* = − 0.4e^−2^, SE = 0.05e^−2^) and for the fixations on the averted gaze area (*β* = − 0.01e^−2^, SE = 0.05e^−2^). That is, as the experiment progressed, neurotypical, but not autistic, participants gazed more at the side of the screen on which the reinforcement would appear.

**Fig. 8:**
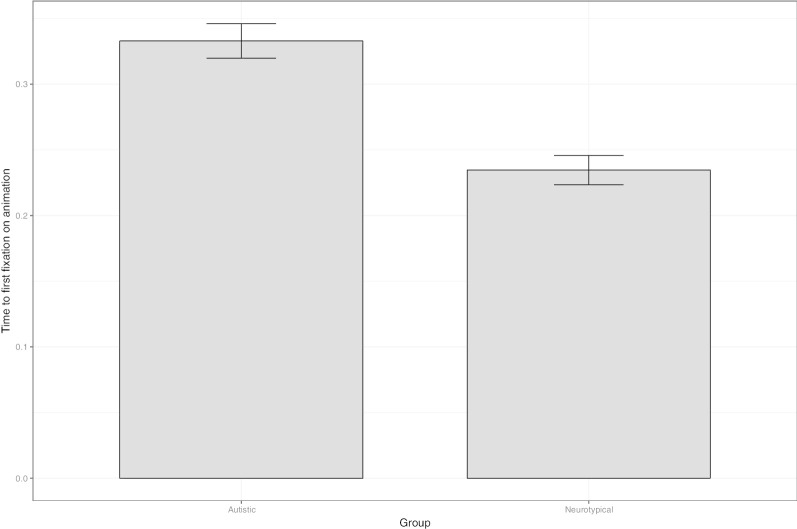
Reinforcement phase. Mean time to first fixation on the animated reinforcement by group. Vertical bars represent standard errors of means

Second, an anticipation of the animated reinforcement should be visible in the timing of the first fixations on the reinforcement video; these are displayed in Fig. 8. Time to first fixation was analysed with multilevel linear regressions, with by item and by participant random intercepts (which was the maximal model to converge). Stepwise comparisons indicated that the addition of group significantly improved the model fit (*χ*^(1)^ = 7.45, *p* = 0.006), as did the addition of trial (*χ*^(1)^ = 6.37, *p* = 0.011) and of the Group × Trial interaction (*χ*^(1)^ = 11.7, *p* < 0.001). Importantly, there was no simple group effect (*p* = 0.86). The simple trial effect indicated that time to first fixation increased over the course of the experiment (*β* = 0.5e^−2^; SE = 0.1e^−2^; *p* < 0.001). However, Group × Trial interaction was due to the fact that the slope was positive in the autistic group (*β* = 0.6e^−2^; SE = 0.1e^−2^), but negative in the neurotypical group (*β* = − 0.1e^−2^; SE = 0.1e^−2^), this latter difference being significant (*p* < 0.001). That is, as the experiment progressed, neurotypical, but not autistic participants, became faster at visually locating the reinforcement animation.

In sum, trial slopes, both in the transition phase and on the first fixations on the animated reinforcement, indicate that an implicit learning of the rule leading to the appearance of the animation took place in the neurotypical, but not in the autistic, group. The neurotypical group was thus sensitive to the reinforcement of the stimulus with the direct gaze.

The absence of reinforcement in the autistic group could be due to the fact that autistic participants were less interested in the animation that constituted the reinforcement. To rule this possibility out, we analysed fixations on the reinforcement illustrations. Stepwise multilevel linear regression on the sum of fixations per trial, with by item and by participant random intercepts, revealed no group effect (*p* = 0.3), indicating that participants in both groups visually explored the reinforcement illustrations to the same extent.

### Gender, social anxiety and alexithymia

**Fig. 9:**
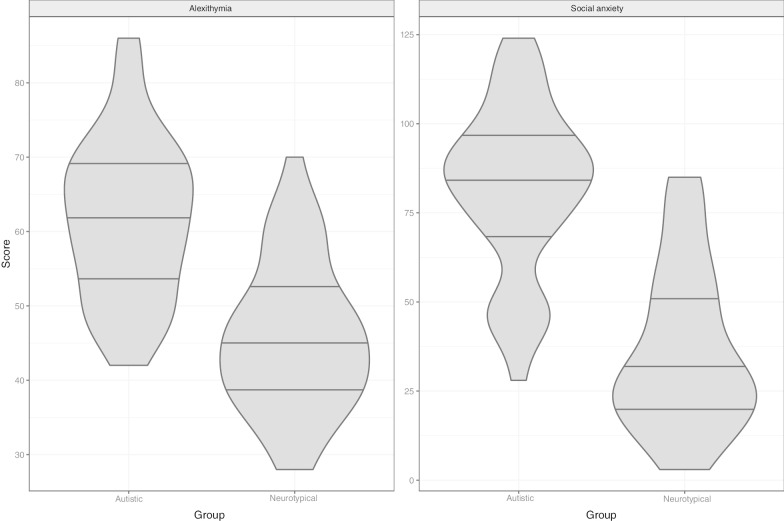
Violin plots for total social anxiety and alexithymia scores per group. Horizontal lines represent 0.25, 0.5 and 0.75 quantiles

As shown in Table 1, our groups did not differ on age (*t* = − 0.101, *df* = 84, *p* = 0.92), full-scale IQ (*t* = − 0.170, *df* = 84, *p* = 0.86), verbal IQ (*t* = 0.581, *df* = 82, *p* = 0.56) and level of education (*t* = 1.636, *df* = 83, *p* = 0.11). As anticipated, however, they differed in terms of economic status (*t* = 3.115, *df* = 83, *p* = 0.002). Group differences on AQ and EQ questionnaires went in the expected direction: no neurotypical participant had an AQ score above 32, and the two groups were significantly different on both measures (AQ: *t* = − 18.006, *df* = 84, *p* < 0.001; EQ: *t* = 11.484, *df* = 84, *p* < 0.001). Finally, the two groups significantly differed on the social anxiety (*t* = − 8.584, *df* = 76, *p* < 0.001) and alexithymia (*t* = − 6.735, *df* = 76, *p* < 0.001) scores. Figure 9 displays the distribution of social anxiety and alexithymia scores per group.

We first assessed the potential role of gender, social anxiety and alexithymia on the proportional fixations on mouth and eye regions, using multilevel linear models with by item and by participant random intercepts and Stimulus Type and the Stimulus Type × Group interaction as fixed factors. Adding gender did not affect the group differences reported above: in the neurotypical group, the amount of fixations on the eye region was still higher than in autistic participants (*β* = 0.05, SE = 0.02; *p* = 0.024) and there was no group difference in proportional fixations on the mouth (*p* = 1). By contrast, once the social anxiety or alexithymia scores were added to the model, the group differences in the amount of fixation on the eye region disappeared (both *p* > 0.91). Running analyses separately for each group revealed that higher alexithymia score predicted a lower proportion of fixations on the eye region in autistic participants (*β* = − 0.5e^−2^, SE = 0.1e^−2^; *p* < 0.001), but not in the neurotypical group (*p* = 0.62). There was a marginal correlation in autistic participants between the social anxiety score and the proportion of fixations on the eye region (*β* = − 0.1e^−2^, SE = 0.58e^−3^; *p* = 0.058), but not in the neurotypical group (*p* = 0.1).

Finally, to control for the potential role of gender, social anxiety and alexithymia on the distribution of visual fixations on stimuli with direct versus averted gaze, we built a multilevel linear model predicting fixation distribution (by 500 ms windows) during stimulus presentation, with by item and by participant random intercepts and Stimulus Type and Stimulus Type × Group interaction as fixed factors. Adding gender again did not affect group differences: in the neurotypical group, the amount of fixations on the stimuli with direct gaze was higher than on the stimuli with averted gaze (*β* = 0.14, SE = 0.6e^−2^; *p* < 0.001), and neurotypical participants fixated more the stimuli with direct gaze than autistic participants (*β* = 0.1, SE = 0.23e^−1^; *p* < 0.001).

By contrast, once the social anxiety or alexithymia scores were added to the model, the group differences in the amount of fixation on the stimuli with direct gaze disappeared (both *p* > 0.12). Running analyses separately for each group revealed that in both groups, a higher alexithymia score predicted fewer fixations on the stimuli with direct gaze (neurotypical group: *β* = − 0.4e^−2^, SE = 0.2e^−2^; *p* = 0.032; autistic group: *β* = − 0.5e^−2^, SE = 0.1e^−2^; *p* = 0.002). The same correlation was found in the neurotypical group for the social anxiety score (*β* = − 0.2e^−2^, SE = 0.08e^−2^; *p* = 0.006), but not in the autistic group (*p* = 0.68).

## Discussion

This is the first study to simultaneously present direct versus averted gaze stimuli to a gender-balanced sample of autistic and neurotypical adults, while controlling for gender, social anxiety and alexithymia. In doing so, we addressed three research questions. First, we asked whether autistic adults would display an atypically high attention to the mouth region when visually exploring speaking faces. Second, we sought to determine how autistic participants react to direct versus averted gaze. Third, we investigated the extent to which gender, social anxiety or alexithymia may account for atypical face processing in autism.

### Gaze patterns: mouths versus eyes

Our results indicate no difference in the allocation of visual attention to the mouth region between autistic and neurotypical participants. Both groups allocated more attention to eyes than to mouths, even though autistic participants looked less at the eye region than neurotypicals. Overall, our results are in line with reports of lower attention to the eyes in autism, with the mouth having no special status [18–21]. This casts doubt on the idea that autistic adults have an intrinsic preference for mouths over eyes [11–17], consistently with previous studies [16, 18–23, 25–30]. Furthermore, because we used speaking stimuli, our results are inconsistent with the idea that autistic adults preferentially gaze at mouths of speaking people because it might provide them with essential interactional information [10]. It is true that the eye region had an increased relevance in our paradigm, as it allowed to anticipate the reinforcement animation. However, our autistic participants did not appear to grasp the link between gaze direction and reinforcement (see below). It is therefore unlikely that the high relevance of the eye region inhibited an intrinsic interest in the mouth region the autistic participants would have otherwise displayed.

### Eye gaze direction: preference, indifference or avoidance

In line with previous studies [39–42, 88], our neurotypical participants displayed a marked preference for direct gaze. By contrast, no preference, either for direct or averted gaze, emerged in our autistic participants, in line with previous studies [42, 88]. Whereas some authors have suggested that autistic individuals have trouble identifying subtly averted gaze [37, 38], in our study, the manipulation between subtle and obvious averted gaze seemed to influence neither the neurotypical participants’ preference for direct gaze nor the absence of any preference in autistic participants.

Importantly, the analyses of fixation data during the transition phase (viz. between the presentation of the stimuli and the appearance of the reinforcement video) showed that, contrary to neurotypicals, autistic adults did not anticipate the reinforcement based on the side on which was displayed the video with direct gaze. They did not increase their fixations on the reinforced side over the course of the trials, and they did not become faster at landing on the reinforcement animation. It seems rather unlikely that the absence of a reinforcement effect in autistic participants is due to a lack of interest in the rewarding animations, as both groups displayed an equal visual exploration of these animations—even though we cannot completely rule this possibility out. Several studies indicate that implicit learning is intact in autism and reinforcement paradigms proved efficient in autistic adults [89, 90]. The absence of a reinforcement effect we observed in the autistic group is, therefore, likely related to a reduced attention to the eye region. Visually exploring the eye region was crucial for discriminating between direct and averted gaze, and hence anticipating the reinforcement animation. Fixation patterns during both the stimulus presentation and the transition phases strongly suggest that our autistic participants did not distinguish between direct and averted gaze. Interestingly, during post-experiment debriefing session, many neurotypical adults asked us how we managed getting such similar videos despite the eye gaze direction difference; conversely, several autistic participants said they did not notice that the two stimuli presented on the screen were different.

Our results contrast with other studies that reported a broadly intact aptitude to detect and use gaze direction in autistic adults [32–35, 91, 92]. This discrepancy could be due the fact that our task was devoid of any explicit instruction as to the visual exploration of the videos. It is possible that our autistic participants did not spontaneously explore the eye region because they assigned less socio-communicative value to the eyes, but that they would have been able to discriminate between direct and averted gaze stimuli if we would have explicitly instructed them to pay attention to the eye region. This interpretation, anchored in the ability–propensity [93, 94] and the compensatory strategies [95, 96] debates, is congruent with three trends in the literature. First, several studies indicate that, contrary to neurotypicals, autistic adults do not have any unconscious preference for direct gaze [40, 42, 88]. Second, the absence of spontaneous processing of social cues in autism is amply documented in the literature and can plausibly be extended to gaze processing [e.g. 97]. Third, several authors argue that the real challenge for autistic people is to allocate social significance to eye cues, rather than processing and discriminating eye gaze direction [98–100].

It is also possible that some autistic participants, instead of being uninterested in the eye region [see 101], experienced hyperarousal in front of stimuli with direct gaze and specifically avoided the eye region in these stimuli [see 102]. Others may also have experienced hyperarousal effects in front of direct gaze earlier in their lives, leading to a reduced propensity to explore the eye region whatever gaze direction is displayed [103, 104]. Further cross-age and physiological studies should investigate the impact of hyperarousal on reduced social attention in autism.

### Individuals’ characteristics: gender, social anxiety and alexithymia

Contrary to our predictions, we found no correlation between gender and any aspect of visual exploration of our stimuli. The few previous studies that found an effect of gender on social processing in autism mainly focused on infants and children [38, 44–49]. The absence of gender effect in our sample thus calls for a developmental perspective on the interaction between gender and social attention in autism.

Scores on a standardized alexithymia questionnaire predicted the preference for direct gaze stimuli in both groups and, consistently with Bird et al. [63], correlated with the amount of fixation on eyes in autistic adults. These results are congruent with the hypothesis that alexithymia is associated with impaired interoception: alexithymic individuals—autistic or not—may experience difficulties in perceiving the internal state of their body, disrupting the processing of social cues and leading to socio-emotional deficits, such as reduced empathy and poor emotion recognition [105–107]. Interoception issues could also account for the fact that in our study, high alexithymia scores were linked to reduced attention to direct gaze stimuli: direct eye gaze seems to induce self-awareness [108, 109] and to enhance interoceptive accuracy [110].

Finally, social anxiety scores accounted for the preference for direct gaze in the neurotypical, but not in autistic, participants. These results are somewhat unexpected, as an impact of social anxiety on eye contact has been documented in both autistic and non-autistic individuals [54–59, but see 60]. We also found a marginal correlation between social anxiety and the amount of attention to the eye region in the autistic group, but not in our neurotypical participants, while most eye-tracking studies have demonstrated a link between social anxiety and visual exploration of faces in non-autistic adults [e.g. 53].

Note that, in comparison with neurotypical participants, autistic participants scored much higher on alexithymia and social anxiety. (53.66% of the autistic adults met the alexithymia cut-off, against 13.51% of the neurotypical group; the 25 quantile social anxiety score in the autistic group is 72, while the 75 quantile score in the neurotypical group is 51.) This imbalance between our groups raises the question whether alexithymia or social anxiety is intrinsically linked to autism [52, 111] or whether they correspond to personal characteristics that may impact attention to eyes in autistic individuals [51, 106].

### Limitations and future directions

The first limitation of our study is that we did not embed our controlled reinforced preferential looking paradigm within a totally naturalistic setting. Investigating actual interactions is clearly an important avenue for better understanding social attention [66], especially as live versus video eye-to-eye contacts seem to evoke different brain responses [112, 113]. (Interestingly, one of our autistic participants told us that he looked at the actors as if they were paintings, instead of actual people.) Second, while we assessed social anxiety through a questionnaire, the reliability of self-reporting in autism may be questioned [114]. Further investigations should complement questionnaires with physiological measures of social anxiety. Third, while our study focused on adults, cross-age research is necessary to gain insight into the developmental course of social attention in autism [see 115, 116]—especially to better understand the potential effects of gender and social anxiety. Fourth, our samples were not balanced in terms of social anxiety and alexithymia profiles: controlling for those variables is crucial, and recruiting participants matched on these dimensions is another challenge for future research. Fifth, the linguistic and intellectual profiles of our autistic participants were within the typical range. It would be interesting to replicate this study in a group of autistic adults with lower linguistic and intellectual profiles. Language abilities could have an impact on visual strategies and social orienting in adults [see 10]. Relatedly, social anxiety might be less prevalent in the autistic population with intellectual delay [52], so that a group of autistic participants with lower verbal and non-verbal IQs could help delineate the influence of social anxiety on social attention in autism.

## Conclusions

Using a novel reinforced preferential looking paradigm, this experiment showed that neurotypical but not autistic adults displayed a marked preference for direct gaze. The absence of preference for direct versus averted gaze in autism is likely due to difficulties in distinguishing eye gaze direction, potentially linked to a reduced spontaneous exploration or an avoidance of the eye region. Social attention and preference for direct versus averted gaze are mediated by alexithymia and social anxiety scores, but not gender.

## Supplementary information

**Additional file 1.** Supplementary details on the structure of the French sentences used in the experiment and on the eye-tracking data correction.

## Data Availability

The datasets generated and analysed during the current study are available from the corresponding author on reasonable request.

## Abbreviations

AQ: Autism quotient
EQ: Empathy quotient
IQ: Intelligence quotient

## Footnotes

1 We use the term ‘autistic people’, rather than ‘people with autism’ or ‘people with Autism Spectrum Disorder’, as this was the terminology explicitly favoured by the majority of our autistic participants [see also 9].
