# Supplementary material for: No preference for direct versus averted gaze in autistic adults: a reinforced preferential looking paradigm
Source: Mol Autism. 2020 Nov 18;11:91. doi: 10.1186/s13229-020-00398-3 (PMC7672906; doi:10.1186/s13229-020-00398-3)
Supplement: Supplementary file 1 — Additional file 1. Supplementary details on the structure of the French sentences used in the experiment and on the eye-tracking data correction. [file 13229_2020_398_MOESM1_ESM.docx]

# Supplementary methods

## **Structure of the French sentences uttered by the actors in the stimulus videos**

The sentences uttered by the actors in the stimulus videos all follow the same structure: [determiner (indefinite) OR pronoun (personal) + noun (singular, masculine or feminine)] = subject + verb (transitive verb, present indicative) + [determiner (indefinite) + noun (singular, masculine or feminine)] = complement 1 + [preposition + determiner (definite or indefinite) OR pronoun (personal) + noun (singular or plural, masculine or feminine)] = complement 2. Example: mon ami montre une photo à son grand-père ('my friend shows a picture to his grandfather'). Gender and number were counterbalanced, as well as the type of determiner. All words were selected according to their high frequency in Lexique3 [1]. Each noun and verb were used only once throughout the sentences. Two independent judges evaluated whether the sentences sounded natural or not. Sentences judged unnatural were excluded.

## **Eye-tracking data correction**

Eye-tracking data can be very sensitive to calibration errors. Even when conditions are rather optimal and calibration is well completed, a lot of factors can influence the quality of the data. For instance, changes in illumination or participant’s fatigue may cause the calibration to degrade as the experiment is running, resulting in a loss of accuracy and precision in the data [2]. In our study, analyses of fixations on the eyes and the mouth required a high degree of precision. In order to increase the reliability of the data that might have undergone a loss in quality, we designed a simple off-line recalibration method.

First, we extracted the unprocessed eye gaze data from Tobii Studio for each participant. In our dataset, this resulted in one column for the horizontal (*x* axis) coordinates of the averaged left and right eye gaze point on the screen in pixels and one column with the same measure for the vertical (*y* axis) coordinates. Then, for each participant, we identified every trial for which there was no hit on the ‘fixation cross’ Area Of Interest during the fixation phase. For each of these problematic trials, we measured the difference in pixels between the *x* and *y* coordinates of the closest fixation to the centre of the cross and the *x* (980 px) and *y* (520 px) coordinates of the centre of the cross.

Deviation X = $Closest X fixation to the cross-980$

Deviation Y = $Closest Y fixation to the cross-520$

Next, in order to make sure that the deviation from the fixation cross was constant, and not due to a random cause, we compared this value between the trial under consideration and the following trial, by always dividing the lowest absolute value by the highest one. This resulted in two correction ratios, one for *x* coordinates and one for *y* coordinates, both between -1 and 1.

Correction ratio X =

$$\left\{ \begin{aligned} \frac{Deviation X trial n}{Deviation X trial n+1} if \left| Deviation X trial n+1 \right|>\left| Deviation X trial n \right| \\ \frac{Deviation X trial n+1}{Deviation X trial n} if \left| Deviation X trial n \right|> \left| Deviation X trial n+1 \right| \end{aligned} \right.$$

Correction ratio Y =

$$\left\{ \begin{aligned} \frac{Deviation Y trial n}{Deviation Y trial n+1} if \left| Deviation Y trial n+1 \right|>\left| Deviation Y trial n \right| \\ \frac{Deviation Y trial n+1}{Deviation Y trial n} if \left| Deviation Y trial n \right|> \left| Deviation Y trial n+1 \right| \end{aligned} \right.$$

For the coordinates of a trial to be eligible for recalibration, correction ratios had to be higher than 0.75, for *x* and *y* independently. Finally, in all trials eligible for recalibration (34%), *x* or *y* coordinates were corrected by subtracting the deviation value of the closest fixation to the cross (i.e. the difference in pixels between the *x* and *y* coordinates of the closest fixation to the centre of the cross and the *x* and *y* coordinates of the centre of the cross) from the original *x* or *y* values (extracted from Tobii Studio).

Corrected X = $Original X fixation-Deviation X$

Corrected Y = $Original Y fixation-Deviation Y$

Once the recalibration had been achieved, fixation data were recoded to determine whether there had been a hit on the different AOIs using the corrected *x* and *y* coordinates of the eye gaze data and the coordinates of these AOIs. As can be seen in **Figure S1**, the correction did not affect the fixation patterns, but did improve precision. Correcting the data did not affect any of the statistically significant results reported in the article.

**Figure S1** Mean proportion of fixation by time point, per Stimulus Type, before and after correction

**References**

1. New B. Lexique 3 : Une nouvelle base de données lexicales. Actes de la Conférence Traitement Automatique des Langues Naturelles (TALN 2006). 2006.

2. Vadillo MA, Street CNH, Beesley T and Shanks DR. A simple algorithm for the offline recalibration of eye-tracking data through best-fitting linear transformation. Behavior Research Methods. 2015;47(4):1365-76.
